# Seroprevalence of *Helicobacter pylori* in Hispanics living in Puerto Rico: A population‐based study

**DOI:** 10.1111/hel.12453

**Published:** 2017-12-06

**Authors:** María González‐Pons, Marievelisse Soto‐Salgado, Javier Sevilla, Juan M. Márquez‐Lespier, Douglas Morgan, Cynthia M. Pérez, Marcia Cruz‐Correa

**Affiliations:** ^1^ University of Puerto Rico Comprehensive Cancer Center San Juan Puerto Rico; ^2^ University of Puerto Rico Medical Sciences Campus San Juan Puerto Rico; ^3^ University of Puerto Rico Rio Piedras Campus San Juan Puerto Rico; ^4^ Vanderbilt University Medical Center Nashville TN USA

**Keywords:** birth cohort effect, *Helicobacter pylori*, *Helicobacter pylori* seropositivity, *Helicobacter pylori* seroprevalence, Hispanic, Population‐based

## Abstract

**Background:**

*Helicobacter pylori* is an important etiologic factor for peptic ulcers and gastric cancer, one of the top ten leading causes of cancer death in Puerto Rico. However, the prevalence of *H. pylori* infections in this population was previously unknown. The aim of this study was to examine the seroprevalence of *H. pylori* and its associated risk factors in Puerto Rico.

**Materials and Methods:**

A cross‐sectional study was designed using an existing population‐based biorepository. Seropositivity was determined using the Premier^™^ *H. pylori* immunoassay. *Helicobacter pylori* seroprevalence was estimated with 95% confidence using marginal standardization following logistic regression. To assess the risk factors associated with *H. pylori* seropositivity, a multivariable log‐binomial model was fitted to estimate the prevalence ratio (PR) and its 95% confidence interval (95% CI).

**Results:**

A total of 528 population‐based serum samples were analyzed. The mean age of the study population was 41 ± 12 years, of whom 55.3% were females. The overall seroprevalence of *H. pylori* was 33.0% (95% CI = 28.3%‐38.1%). Increasing age and having <12 years of education were significantly (*P* < .05) associated with *H. pylori* seropositivity in the multivariable model; however, residing in counties with low population density reached marginal significance (*P* = .085).

**Conclusions:**

We report that *H. pylori* infection is common among Hispanics living in Puerto Rico. The *H. pylori* seroprevalence observed in Puerto Rico is similar to the seroprevalence reported in the overall population of the United States. The association between *H. pylori* seroprevalence and the risk factors analyzed offers insight into the epidemiology of gastric cancer in Puerto Rico and warrants further investigation.

## 1 INTRODUCTION

*Helicobacter pylori* is a bacterial species responsible for the most common chronic bacterial infection worldwide; close to 50% of the world's population is infected.^1^ *Helicobacter pylori* is strongly associated with chronic gastritis, peptic ulcers, and gastric cancer.^2^ Approximately 10%‐15% of *H. pylori‐*infected individuals develop peptic ulcers and 1%‐2% develop gastric adenocarcinomas.^3^, ^4^ Gastric cancer is the most common infection‐associated cancer worldwide, which led to the classification of *H. pylori* as a class I human carcinogen by the International Agency for Research on Cancer (IARC).^5^ In the United States (US), there are marked racial/ethnic disparities in gastric cancer, with higher incidence and mortality rates among Hispanics and other racial/ethnic groups compared to non‐Hispanic Whites.^6^

Substantial variability in *H. pylori* prevalence is observed globally according to geographic location, age, and socioeconomic status. A birth cohort effect has been observed in *H. pylori* prevalence in most of the world.^7^, ^8^ The Centers for Disease Prevention and Control (CDC) estimate that the prevalence of *H. pylori* infection in developing countries is 70% and is 30%‐40% in most industrialized countries. In the US, the prevalence of *H. pylori* infection is approximately 31%, with variable prevalence among diverse racial/ethnic groups: 21% in Whites, 52% in African Americans, and 64% in Mexican Americans.^6^, ^9^ Marked differences in *H. pylori* infection have also been reported among Hispanic individuals from different regions of Central and South America.^10^, ^11^, ^12^ Individuals with low levels of education and low socioeconomic status have reported to have more than 5.5‐ and 6.6‐times higher odds of being infected with *H. pylori*.^13^

Gastric cancer is the fifth most common incident cancer and the third leading cause of cancer death worldwide.^14^ Gastric cancer incidence rates vary dramatically across regions and countries and are higher in less developed countries. In general, gastric cancer is more common in East Asia, Eastern Europe, and the mountainous regions of Latin America.^15^, ^16^ In Puerto Rico during 2008‐2012, gastric cancer was the fifth leading cause of cancer death for men and the eighth cause for women.^17^ The age‐standardized incidence and mortality rates (per 100 000 population) were 9.0 and 6.1 for males, and 5.2 and 3.5 for females, respectively.^16^ The specific risk factors associated with gastric cancer, including the prevalence of *H. pylori* infection among Hispanics living in Puerto Rico, are currently unknown. The aim of this study was to examine the seroprevalence of *H. pylori* and its associated risk factors in Hispanics living in Puerto Rico using samples from a population‐based biorepository.

## 2 METHODS

### 2.1 Study design and population

We used a representative sample from an existing population‐based biorepository of archived, frozen serum samples (n = 1645) from a seroepidemiologic survey of viral hepatitis and other infections in Puerto Rico. The study design has been previously described by Pérez et al.^18^ In this study, a secondary analysis was performed using an estimated sample size of 528 subjects, which assumed a conservative *H. pylori* seroprevalence. In brief, the parent seroepidemiologic study “*Epidemiology of Hepatitis C in the Adult Population of Puerto Rico”* aimed to recruit noninstitutionalized individuals aged 21‐64 years old residing in Puerto Rico at the time of the survey (2005‐2008). This study used a stratified, multistage, and probability cluster design of all households in Puerto Rico. The data collection instrument designed for this study was modeled after questionnaires previously used in household studies in Puerto Rico. The questions were based on the questionnaire developed by the ARIBBA (Alliance for Research in El Barrio, New York, and Bayamón) Project, a study that evaluated factors that influenced risk behaviors among Puerto Rican injection drug users.^19^ Two field managers visited every selected occupied household, where subjects that agreed to participate in the study completed the following procedures:^1^ informed consent and pretest counseling;^2^ a personal interview to obtain information about sociodemographic characteristics, medical history, tattooing and body piercing practices, knowledge on viral infections, and self‐report of hepatitis A and hepatitis B vaccination;^3^ an audio computer‐assisted self‐interview to ascertain cigarette and alcohol use, drug use, sex‐related risk behaviors, and history of incarceration; and^4^ collection of blood and urine samples. Of the selected residents, 1654 (77.9%) participated in the study. Although the age distribution of the sample was similar to that of the adult population of Puerto Rico according to the Census 2000, females were slightly overrepresented as they made up 56.4% of the study sample.

The secondary analysis in this study was performed using an estimated sample size of 528 subjects, which assumed a conservative *H. pylori* seroprevalence of 20%, a precision of 3.5%, 95% confidence, and a sample loss of 5%. Serum samples were analyzed using the Premier^™^ *H. pylori* enzyme immunoassay (Meridian Bioscience, Inc. Cincinnati, OH, USA) according to the manufacturer's specifications. This immunoassay was specifically designed for the *in vitro* qualitative detection of *H. pylori* IgG antibodies in human serum and plasma samples with a relative sensitivity of 99.2% and a relative specificity of 96.0%.

### 2.2 Study variables

Sociodemographic and hygienic risk factors for *H. pylori* seropositivity were identified from the literature.^6^, ^20^, ^21^ However, only the following variables were available for analysis in the parent study database: sex (male vs female), age group in years (21‐29, 30‐49, and 50‐64), marital status (married/consensual partner, never married, or divorced/separated/widowed), educational attainment in years (<12 vs ≥12), and adult population density of in county of residence (low defined as equal to or below median population density vs high defined as above median population density). The median population density was defined as 486 adults per square mile in the parent study.^18^

### 2.3 Statistical analysis

Prevalence of *H. pylori*, overall and by sociodemographic characteristics, was estimated using marginal standardization following logistic regression.^22^ Due to the complex sampling design, the prevalence estimation, and the simple and multivariate log‐binomial models were weighted according to the probability of participation in each household block and the inverse of the probability of selection according to the geographic strata, household blocks, and sex distribution according to postcensal estimates in Puerto Rico. Simple log‐binomial models were fitted to estimate the unadjusted prevalence ratio (PR) and its 95% confidence interval (CI) associated with each independent variable. The PR was used in this analysis as a measure of association because it is considered more conservative, consistent, and appropriate for cross‐sectional studies compared to the prevalence odds ratio.^23^, ^24^ Variables were entered into the multivariate log‐binomial model if the variables were known or hypothesized risk factors for *H. pylori*, and the *P* values associated with their regression coefficients were <.05.^25^ All data were analyzed using Stata for Windows release 14.0 (Stata Corporation, College Station, TX, USA).

## 3 RESULTS

### 3.1 Prevalence of *H. pylori*, overall and by sociodemographic characteristics

The mean age of the study participants from which the selected serum samples were collected in the parent study was 41 ± 12 years, of whom 55.3% were females (Table 1). The majority of participants were married, had at least 12 years of education, and lived in low‐density populated counties in Puerto Rico. The overall seroprevalence of *H. pylori* in this cohort was 33.0% (95% CI: 28.3%‐38.1%). *Helicobacter pylori* seroprevalence increased significantly with age; the highest seroprevalence (44.3%) was observed among participants 50‐64 years old. A comparable seroprevalence was observed among men and women (35.7% and 30.9%, respectively). Higher *H. pylori* seroprevalence was detected among participants with <12 years of education (46.2%) and those that lived in low‐density populated counties (41.0%).

**Table 1:** Seroprevalence of *Helicobacter pylori,* overall and by sociodemographic characteristics

Characteristic	n (%)	Seroprevalence (%) (95% CI)
Overall	528	33.0 (28.3‐38.1)
Age group in years		
21‐29	118 (22.4)	21.1 (14.5‐29.6)
30‐39	124 (23.5)	22.6 (14.9‐32.7)
40‐49	141 (26.7)	41.3 (31.7‐51.7)
50‐64	145 (27.5)	44.3 (35.3‐53.6)
Sex		
Female	292 (55.3)	30.9 (25.1‐37.3)
Male	236 (44.7)	35.7 (28.4‐43.6)
Marital status		
Never married	111 (21.0)	22.4 (15.5‐31.3)
Married/consensual partner	307 (58.1)	35.0 (29.5‐41.0)
Divorced/separated/widowed	110 (20.8)	39.2 (28.7‐50.9)
Years of education		
≥12	403 (76.3)	29.4 (24.3‐35.2)
<12	125 (26.7)	46.2 (35.9‐56.9)
Population density of residential county^a^		
Low	252 (47.7)	41.0 (34.6‐47.7)
High	276 (52.3)	29.4 (23.5‐36.1)

a Defined as low if the adult population density is equal to or below the median (486 adults per square mile) vs high if above the median.

### 3.2 Risk factors associated with *H. pylori* seropositivity

*Helicobacter pylori* seroprevalence significantly increased with age (Table 2); however, after multivariable adjustment for marital status, years of education, and population density of current county of residence, the prevalence of *H. pylori* remained significantly higher only among those aged ≥40 years relative to those aged 21‐29 years (PR_40‐49_ = 1.8 (95% CI = 1.1‐2.9); PR_50‐64_ = 1.7 [95% CI = 1.0‐2.9]). The prevalence of *H. pylori* seropositivity was also significantly higher among those with <12 years of education and low population density in their residential county. After multivariable adjustment, these prevalence ratios were attenuated, but remained significant (PR = 1.4, 95% CI = 1.0‐1.8) and marginally significant (PR = 1.3 95% CI = 1.0‐1.6), respectively. Although *H. pylori* seroprevalence was significantly (*P* < .05) associated with marital status in the simple regression model, the prevalence ratio was attenuated and was not significant in the multivariable model.

**Table 2:** Prevalence ratio (PR) of *Helicobacter pylori* seropositivity according to sociodemographic characteristics

Characteristic	PR_unadjusted_ (95% CI)	*P*‐value	PR_adjusted_ a (95% CI)	*P*‐value
Age group in years				
21‐29	1.0		1.0	
30‐39	1.1 (0.6‐1.9)	.847	1.0 (0.6‐1.8)	.994
40‐49	2.0 (1.2‐3.2)	.007	1.8 (1.1‐2.9)	.028
50‐64	2.1 (1.3‐3.4)	.002	1.7 (1.1‐2.9)	.035
Marital status				
Never married	1.0		1.0	
Married/consensual partner	1.6 (1.0‐2.5)	.057	1.3 (0.8‐2.2)	.215
Divorced/separated/widowed	1.8 (1.1‐3.0)	.029	1.4 (0.8‐2.3)	.245
Years of education				
≥12	1.0		1.0	
<12	1.6 (1.2‐2.1)	.002	1.4 (1.0‐1.9)	.048
Population density of residential county^b^				
High	1.0			
Low	1.4 (1.1‐1.8)	.014	1.3 (1.0‐1.6)	.085

a Adjusted by all variables in the model.

b Defined as low if adult population density is equal to or below the median (486 adults per square mile) vs high if above the median.

## 4 DISCUSSION

*Helicobacter pylori* is a major risk factor for the development of gastric cancer, the third leading cause of cancer death worldwide.^14^ In Puerto Rico, gastric cancer is one of the top ten leading causes of cancer death^17^; however, the prevalence of *H. pylori* infection among Hispanics living in Puerto Rico is currently unknown. In this study, we report the first seroprevalence estimates of *H. pylori* infection and explored its association with sociodemographic factors among a representative population‐based sample of Hispanics living in Puerto Rico. The information revealed by our study will contribute to a better understanding of the subgroups at higher risk of *H. pylori* infection in Puerto Rico and should serve as a guide for future research, as well as to better tailor gastric cancer prevention and control strategies for this minority population.

The *H. pylori* seroprevalence (33.0%) observed among our cohort of Hispanics living in Puerto Rico is comparable to the percentage reported in the US, where the prevalence of *H. pylori* infection is approximately 30.7%.^6^ However, the seroprevalence detected in our cohort was significantly lower to the *H. pylori* seroprevalence reported among Mexican Americans (64.0%) in a study analyzing data from the National Health and Nutrition Examination Survey (NHANES), which determined seropositivity status using a comparable, commercial enzyme immunosorbent assay.^6^ This study reports that Mexican Americans and non‐Hispanic Blacks (52%) had a significantly higher seroprevalence of *H. pylori* when compared to non‐Hispanic Whites even after adjusting for country of origin (not US born vs US born) and socioeconomic status.^9^ In Central and South America, *H. pylori* seroprevalence, as determined by ELISA, has been reported to range from 50.7% to 84.7% depending on the region.^12^, ^26^, ^27^, ^28^ The observation that the seroprevalence detected among Hispanics living in Puerto Rico is more similar to estimates in general population from the US, rather than estimates from other Latin American countries, may be attributed to the US (and Puerto Rico as a US territory) having more developed living conditions compared to less developed regions in Latin America that have high *H. pylori* seropositivity, such as Honduras and Nicaragua.^29^ In addition, it is important to note that since Puerto Rico is a US territory, the same guidelines (e.g. American College of Gastroenterologists) to test for and treat *H. pylori* are used by physicians in both Puerto Rico and the US.^30^ Also, increased incidental antibiotic use could possibly explain why *H. pylori* seropositivity levels in the US and in Puerto Rico are comparable and lower than seropositivity levels reported in other Latin American countries. In a study reporting worldwide antibiotic usage, individuals in the US were found to use more antibiotics than Latin American countries.^31^, ^32^

After multivariable adjustment, only age and years of education remained significantly associated with *H. pylori* seroprevalence. Although *H. pylori* seroprevalence was not significantly associated with sex in the bivariate‐ or multivariable‐adjusted models, increasing age was significantly associated with *H. pylori* seroprevalence in our study population. Various studies have reported that sex is not associated with increased risk of *H. pylori* infection.^33^, ^34^, ^35^ In industrialized countries, the prevalence of *H. pylori* infection is low in childhood and slowly increases with age.^2^ Several groups have reported increasing *H. pylori* seroprevalence according to age, and it is hypothesized that this increase is attributable to a birth cohort phenomenon. However, the cross‐sectional nature of the present study limits our ability to determine whether the increased prevalence of *H. pylori* with age is due to a cohort effect. Although increased incidental use of antibiotics may in part explain the decreasing trend of *H. pylori* seroprevalence in younger individuals in our study, future studies are needed to fully understand the factors contributing to the associations observed.

Prevalence of *H. pylori* seropositivity was also significantly higher among individuals with low educational levels (<12 years of education). Associations between low educational level and *H. pylori* seropositivity have been previously documented.^36^, ^37^, ^38^ Using data from NHANES III (1988‐1994) and NHANES 1999‐2000, Grad et al.^6^ reported significantly higher odds of *H. pylori* infection as education level decreased. In NHANES 1999‐2000, participants without a high school education had significantly higher odds of *H. pylori* infection compared to participants that had attended college (OR = 4.87, 95% CI: 3.66‐6.47). Education has been widely used as a surrogate marker of socioeconomic status given its stability over the adult lifespan, and its association with morbidity and mortality.^39^

After multivariable adjustment, we observed a marginally significant association between *H. pylori* seroprevalence and low population density. In Puerto Rico, population density can be related to social inequalities such as poverty. According to the 2010 US Census Bureau, high‐density areas tend to be urban areas with lower levels of poverty (44%) and land values, whereas low‐density areas tend to be rural and have higher levels of poverty (56%).^40^ Furthermore, our group found a significant inverse correlation (*r* = −.63, *P* < .0001) between population density and poverty level when analyzing the 2010 US Census Bureau data on the Puerto Rican population. Thus, the significant association between *H. pylori* seroprevalence and having <12 years of education, and the marginally significant association observed among individuals residing in low population density counties, supports that low socioeconomic status may be a risk factor for *H. pylori* infection in Puerto Rico. However, further research studies are necessary to determine the factors associated with *H. pylori* infection, with special attention to socioeconomic indicators.

A limitation in our study to consider is that the data collection instruments used in the parent study were designed to assess risk factors associated with hepatitis C and other viral hepatitis in adults living in Puerto Rico.^18^ Therefore, information that would have contributed to a better understanding of additional risk factors associated with *H. pylori* seropositivity in Puerto Rico, such as county of residence in order to determine altitude, previous *H. pylori* treatment, if individuals had previously resided outside of Puerto Rico for any given period of time, water source, diet, living conditions, and occupation, were unavailable. Although the distribution of age and education level in our sample was comparable to that of the adult population of Puerto Rico, according to the 2000 US Census, females were overrepresented. However, prevalence of *H. pylori* study was not affected when the corresponding proportions of males and females in Puerto Rico from the Census 2000 were considered. Moreover, children, adolescents, and homeless individuals were excluded from the parent study, limiting the generalizability of study findings. Although race has been associated with *H. pylori* seropositivity,^9^ due to the fact that Puerto Rican Hispanics are a highly racially and genetically admixed population,^41^ and that questions that address race are currently not used in epidemiological studies in Puerto Rico, we did not examine race as a risk factor for *H. pylori* seropositivity on the island. Epidemiological studies examining the Puerto Rican population currently do not tend to address race in their data collection instruments based on the results of focus groups comprised of Census responders in Puerto Rico who unanimously agreed that the question on race was inappropriate for Puerto Ricans when this question was introduced into the Census questionnaire in Puerto Rico in 2000.^42^ Participants felt the question was racist and most reluctantly answered the question based on skin color, which is considered an inadequate definition of race. The strength of this study is based on the systematic evaluation of serum samples to determine *H. pylori* seropositivity in representative subsample taken from a well‐characterized, population‐based biobank of Hispanics living throughout Puerto Rico.^18^

In conclusion, the seroprevalence reported in this study provides baseline information regarding *H. pylori* infection among Hispanics living in Puerto Rico and will serve as a basis for future studies of *H. pylori*‐associated diseases, such as peptic ulcer disease and gastric adenocarcinoma. The *H. pylori* prevalence observed among this cohort may have an impact in the incidence of gastric neoplasia and may contribute to a disproportionate cancer burden among individuals with low socioeconomic status. Additional epidemiological studies have even more relevance in the aftermath of the hurricane Maria in Puerto Rico^43^ and the possibility of an increase of *H. pylori* infections after this natural disaster. Infectious disease outbreaks are a major concern after a natural disaster given the limited access to food and safe drinkable water, overcrowded shelters, poor hygiene practices, exposition to wastewater, and inadequate access to medical care.^44^ Studies have described an increase in *H. pylori* infections among individuals who survived natural disasters.^45^ Therefore, a better understanding of the risk factors associated with *H. pylori* infection in this Hispanic population will be of the utmost importance given the established association between *H. pylori* and gastric cancer, and the high burden of this malignancy in Puerto Rico.

## DISCLOSURES

No author has conflicts of interest or financial arrangements that could potentially influence the described research. Competing interests: the authors have no competing interests.
